# Resistance against Integrase Strand Transfer Inhibitors and Relevance to HIV Persistence

**DOI:** 10.3390/v7072790

**Published:** 2015-07-07

**Authors:** Thibault Mesplède, Mark A. Wainberg

**Affiliations:** 1 McGill University AIDS Centre, Lady Davis Institute for Medical Research, Jewish General Hospital, Montréal, QC H3T 1E2, Canada; 2 Division of Experimental Medicine, Faculty of Medicine, McGill University, Montréal, QC H3A 2R7, Canada; 3 Department of Microbiology and Immunology, Faculty of Medicine, McGill University, Montréal, QC H3A 0G4, Canada

**Keywords:** HIV, integrase strand-transfer inhibitors, resistance, raltegravir, elvitegravir, dolutegravir, R263K, viral fitness, viral reservoirs, HIV eradication

## Abstract

Drug resistance prevents the successful treatment of HIV-positive individuals by decreasing viral sensitivity to a drug or a class of drugs. In addition to transmitted resistant viruses, treatment-naïve individuals can be confronted with the problem of drug resistance through *de novo* emergence of such variants. Resistant viruses have been reported for every antiretroviral drug tested so far, including the integrase strand transfer inhibitors raltegravir, elvitegravir and dolutegravir. However, *de novo* resistant variants against dolutegravir have been found in treatment-experienced but not in treatment-naïve individuals, a characteristic that is unique amongst antiretroviral drugs. We review here the issue of drug resistance against integrase strand transfer inhibitors as well as both pre-clinical and clinical studies that have led to the identification of the R263K mutation in integrase as a signature resistance substitution for dolutegravir. We also discuss how the topic of drug resistance against integrase strand transfer inhibitors may have relevance in regard to the nature of the HIV reservoir and possible HIV curative strategies.

## 1. Introduction

The rapid emergence of HIV drug resistance was first observed in individuals treated with a single antiretroviral molecule (monotherapy) and led to the implementation of highly active antiretroviral therapy (HAART) that classically combines three antiretroviral drugs for the treatment of HIV-positive individuals [1]. HAART precludes the emergence of drug resistance in most individuals. However, viral strains from some individuals who are not successfully treated with HAART can develop resistance mutations, independently of whether these individuals are treatment-experienced or treatment-naïve. Treatment-selected resistant strains have been documented against inhibitors of each of reverse transcriptase (RT), protease (PR), fusion, and integrase as well as against CCR5 receptor antagonists [2]. The clinical relevance of drug resistance can be interpreted through the use of genotypic resistance algorithms that can inform treatment decision-making for individuals who live with resistant HIV viruses.

**Figure 1:**
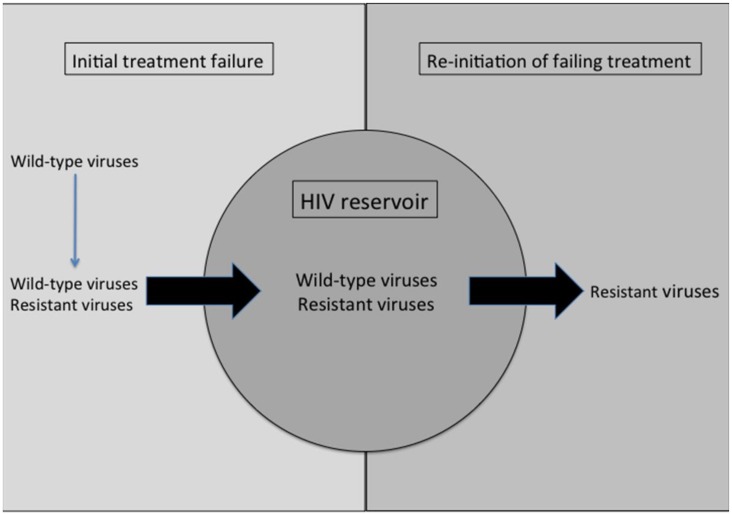
HIV resistant viruses are archived within the viral reservoir upon treatment failure and can rapidly reemerge following treatment re-initiation.

Although treatment cessation usually results in reversion of resistant viral populations to wild-type [3], treatment re-initiation commonly leads to the rapid re-occurrence of drug resistance [4], effectively precluding the future use of antiretroviral drugs that may have been compromised by resistance mutations that arose during initial treatment failure. This is due to the rapid archiving of drug resistant viruses concomitantly with their emergence under treatment pressure [2]. Interestingly, these observations demonstrate that HIV long-lived viral reservoirs can be fed by active replication that occurs during acute HIV infection and that at least part of the viral reservoir that is constituted under these circumstances is persistently replicating and/or can be spontaneously reactivated from latency (Figure 1).

The most recent antiretroviral drugs to have been approved for therapy are integrase strand transfer inhibitors (INSTIs). These inhibitors include raltegravir (RAL), elvitegravir (EVG) and dolutegravir (DTG), each of which has been shown to be well-tolerated with relatively few side effects: this is perhaps due to the fact that there is no human enzyme that is functionally homologous to HIV integrase, in contrast to RT and PR. In addition to their low toxicity, INSTI-based antiretroviral regimens are highly efficacious at suppression of HIV replication *in vivo* and are now recommended for initiation of HIV therapy in adults [5,6,7,8,9]. In rare instances, HIV can become resistant against INSTIs through the emergence of discrete mutations within the integrase coding region. Those resistance substitutions have been reviewed elsewhere [10,11,12,13,14,15]. The object of the current review is to discuss the emergence of HIV resistant viruses in individuals treated with INSTIs and how data obtained with DTG may relate to HIV reservoirs and the potential to achieve viral eradication.

## 2. Resistance against Raltegravir

Raltegravir is recommended at a dose of 400 mg twice daily and when used together with two nucleoside drugs has been shown to be non-inferior over three years to a regimen composed of efavirenz (EFV), tenofovir (TDF) and emtricitabine (FTC) and superior after that [9,16,17,18,19,20]. Resistance mutations that were found in viral isolates from treatment-naïve participants who experienced treatment failure during the initial dose-ranging Protocol 004 clinical trial were: L74L/M, V151I, N155H, Y143R and S230R in integrase (IN) and M184M/I/V and K65K/R in RT [18] (Table 1). M184I/V were the most common resistance mutations in this study. The virus from one of the individuals who experienced RAL-based treatment failure was found to possess only the M184V resistance substitution, in the absence of any mutation in the integrase coding sequence, whereas the other viruses were found to be resistant against both integrase and RT inhibitors [18]. In particular, the combination of N155H in integrase with M184M/I/V in reverse transcriptase was commonly observed [18]. Similar results were observed during the STARTMRK clinical trial, in which viral isolates from treatment-naïve participants who experienced RAL-based treatment failure developed resistance mutations, mostly against both INSTIs and reverse transcriptase inhibitors [9,18,19]. Treatment failure was also associated with the emergence of variants that were resistant solely against either INSTIs or RT inhibitors [9,18,19]. When the protease inhibitor darunavir (DRV) was used in combination with RAL in the NEAT/ANRS143 clinical trial, only the N155H resistance mutation in integrase was found, in the absence of any mutation in PR [21]. This observation is in agreement with the fact that DRV possesses a higher genetic barrier for resistance than nucleos(t)ides RT inhibitors (NRTIs) that were used in the Protocol 004 and NEAT studies. The rapid archiving of resistant strains against raltegravir has also been documented [22].

**Table 1:** Examples of new IN and RT drug resistant mutations emerging after treatment failure with raltegravir.

Clinical Trial	Integrase	Reverse Transcriptase	Type of Participant
Protocol 004 (week 96)	L74L/M, V151I, N155H	M184M/I/V, K65K/R	Treatment-naive
	7143C, S130R	M184M/I/V	
	N155H	M184M/I/V	
	-	M184M	
NEAT001 (week 96)	N155H	-	Treatment-naive
	N155H	-	
	N155H	-	
	N155H	-	
	N155H		
	-	K65R	
STARTMRK (week 144)	G140S, Q148H	M184I/V in 3 cases	Treatment-naive
	G140S, Q148R		
	L74L/M, E92Q, T97A, Y143H, Y143R		
	-	Other mutations in 3 cases	
	-		
	-		

## 3. Resistance against Elvitegravir

Elvitegravir boosted by cobicistat and in combination with TDF/FTC has demonstrated superiority to EFV/TDF/FTC or ritonavir-boosted atazanavir (ATV/r) [23,24,25,26,27]. EVG is also non-inferior to RAL in highly treatment-experienced individuals [28,29]. Similar to RAL, treatment failure with EVG-based therapy in treatment-naive individuals has been correlated with the development of resistance against both EVG and the NRTIs that were co-administered with it (Table 2). Under such circumstances, extensive genotypic characterisation of resistant strains from two large clinical studies, *i.e.*, Studies 102 and 103, revealed the emergence of the T66I, E92Q, T97A, Q148R and N155H resistance mutations in integrase, mostly in combination with the M184I/V substitutions in RT [23,26,27,30,31,32]. Less frequently, the K65R substitution in RT was found in combination with M184I/V and integrase resistance mutations. In regard to integrase substitutions, considerable overlap was observed among the resistance mutations that emerged after treatment failure involving either RAL or EVG. This points to the issue of extensive cross-resistance between these two compounds, precluding the sequential use of these drugs in the clinic [14,15]. It is also important that Studies 102 and 103 showed that RT resistance substitutions emerged before integrase resistance mutations in 4 of 13 viral isolates from which both the integrase and RT coding regions could be sequenced at the time of initial treatment failure as well as at subsequent time points [33]. In these 13 individuals, no virus was found to develop integrase resistance mutations in the absence of pre-existing or concomitant RT resistance substitutions.

**Table 2:** Examples of new IN and RT drug resistant mutations emerging after treatment failure with elvitegravir.

Clinical Trial	Integrase	Reverse Transcriptase	Type of Participant
Studies 102 and 103 (week 144)	-	M184V	Treatment-naive
	-	M184V/I, K65K/R	
	E92Q	M184V	
	T66T/I, E92E/Q, N155N/H, E157E/Q	ND	
	N155H	M184V	
	E92Q	M184I	
	Q148R	M184V	
	E92Q, S153A	M184V, K65K/R	
	E92E/Q, Q1481/R, N155H/N	M184V	
	G140C, Q148R	M184V, K65R, A62A/V	
	E92Q, H51H/Y, L68V	M184V, K65R, A62A/V	
	T66T/I, E92E/1	M184V	
	E92Q	M184V	
	E92Q	M184V	
	N155H	M184V	
	N155H	M184V, K65R, A62A/V	
	T97A, G163G/R	M184V	
	ND	M184M/V	

## 4. Resistance against Dolutegravir

DTG is recommended at 50 mg QD for adults who have not been previously exposed to other INSTIs and does not require the use of a boosting agent to maintain durable efficacious daily concentrations. DTG can also be used as part of a single tablet regimen in combination with abacavir (ABC) and lamivudine (3TC). This DTG/3TC/ABC combination was shown to be superior to EFV/TDF/FTC for the treatment of treatment-naïve individuals, in large part because of a larger number of serious adverse events in the EFV arm [34,35]. DTG was also superior to DRV/r when either drug was used in combination with two NRTIs [36]. DTG was also shown to be non-inferior to RAL, both in association with two NRTIs, at suppressing viral replication [37,38].

In regard to resistance, DTG seems to be unique amongst all antiretroviral drugs, including INSTIs, because of no *de novo* resistance mutation, either in regard to DTG itself or the NRTIs with which it has been co-administered, has ever been reported in previously treatment-naïve individuals (Table 3) [34,36,37,38]. This observation is specific for treatment-naïve individuals.

Two scenarios need to be considered in regard to treatment-experienced persons and the occurrence of resistance against DTG. First, many treatment-experienced individuals who have been treated with DTG as part of second-line therapy, after previously failing either RAL or EVG, have developed new INSTI-related resistance mutations [39]. This was true even though the dose of DTG in the aftermath of prior failure with either RAL or EVG was doubled to 50 mg twice daily. In this setting, the likelihood of therapeutic success with DTG-based therapy was far less than in first-line treatment because of the pre-existence of INSTI mutations that confer cross-resistance among each of DTG, RAL, and EVG [39,40,41].

**Table 3:** Examples of new IN and RT drug resistant mutations emerging after treatment failure with dolutegravir.

Clinical Trial	Integrase	Reverse Transcriptase	Type of Participant
Spring-1 (week 48)	-	M184M/V (DTG 10 mg)	Treatment-naive
Spring-2 (week 96)	-	-	Treatment-naive
Single (week 48)	-	-	Treatment-naive
Flaminga (week 48)	-	-	Treatment-naive
Saling (week 48)	R263K	-	Treatment-experienced but INSTI-naive
	R263R/K	-	
	V151I/V	-	
	T97A/E138T/A	-	

The second scenario in which DTG has been used to treat treatment-experienced persons has involved patients who had failed multiple drugs but whom had never before been treated with an INSTI [42]. In this scenario, the likelihood of therapeutic success with DTG is higher than in RAL- or EVG-experienced patients, probably because these patients would not be expected to possess any INSTI-related mutations. The study that examined this was termed SAILING and compared the use of RAL *versus* DTG, both together with genotypically-directed optimum background therapy, and showed that DTG was superior to RAL in this context. In this study, the patients who experienced RAL-based treatment failure developed an array of well-described INSTI mutations that are known to be associated with this drug. In contrast, very few patients in the DTG arm developed new drug resistance although the viral isolates from two individuals with protocol-defined virological failure (PDVF) after 24 weeks of treatment were found to have developed a R263K integrase substitution or a R263K/R mixture [42]. Both of these individuals were still unsuppressed at week 48 and genotyping at this time revealed that the virus had not developed additional mutation compared to week 24. Nor did the R263K/R mixture further evolve towards a pure R263K population. Consistent with these results, the levels of resistance against DTG that are associated with these changes did not increase between weeks 24 and 48, *i.e.*, <2-fold for both isolates at both time points. Two additional DTG-treated individuals met the criteria for PDVF at week 48. One of them possessed an integrase V151V/I mixed population that was not phenotypically resistant against DTG while the other possessed substitutions at positions T97A and E138T/A and was shown to have high levels of resistance against DTG [42]. However, virus isolates from the latter individual were shown to have a pre-existing Q148 mutation at the time of enrolment, suggesting that this individual had not, in fact, been INSTI-naïve. Since the participants in this clinical trial were heavily treatment-experienced individuals with limited treatment options, these findings suggest that DTG alone is insufficient to completely inhibit HIV replication *in vivo*.

The presence of the R263K substitution (2/4) amongst viral isolates from individuals who experienced PDVF in this study was in agreement with previous work that had selected R263K in tissue culture with DTG [43] over 20 weeks. Site-directed mutagenesis confirmed that R263K confers low-level resistance to DTG and work with recombinant integrase enzyme containing R263K showed that this substitution decreases the affinity of the integrase protein for DNA and its ability to perform strand transfer. Tissue culture assays with viruses containing R263K revealed that this mutation compromised each of viral infectivity, viral replicative capacity, and integration of viral DNA in both cell lines and in primary human peripheral mononuclear cells (PBMCs) [43,44]. The growth of R263K-containing viruses in tissue culture for more than four years has not yet led to the identification of any additional compensatory substitution that might be able to reverse the consequences of R263K. This is important since compensatory mutations are commonplace in the aftermath of primary drug resistance substitutions and function both to restore viral replication capacity and enzymatic function and to simultaneously increase the levels of drug resistance, which is why such substitutions are selected to begin with. Although substitutions at positions M50I, H51Y or E138K emerged under DTG pressure after R263K, none of these was able to restore viral replicative capacity to wild-type levels [44,45,46]. Nor were any other secondary or tertiary mutation subsequently found [47]. Moreover, R263K also inhibited the ability of HIV to develop of resistance against RT inhibitors such as 3TC [48], an observation that helps to explain the excellent clinical trial results obtained to date with DTG.

In contrast to RAL or EVG, no NRTI resistance was detected in either treatment-naïve or treatment-experienced individuals who experienced DTG-based treatment failure when the latter drug was co-administered with RT inhibitors [34,36,37,38,42]. This result might also be explained by the fact that a further decrease in viral replicative capacity occurs when the R263K substitution in integrase and the M184I/V substitutions in RT are combined in the same virus, compared to the presence of either substitution alone [49]. In addition, exposure of HIV in tissue culture to DTG compared to RAL may more efficiently limit HIV genetic diversification [50].

## 5. Implications of HIV Non-Resistance against Dolutegravir for the HIV Reservoir

**Figure 2:**
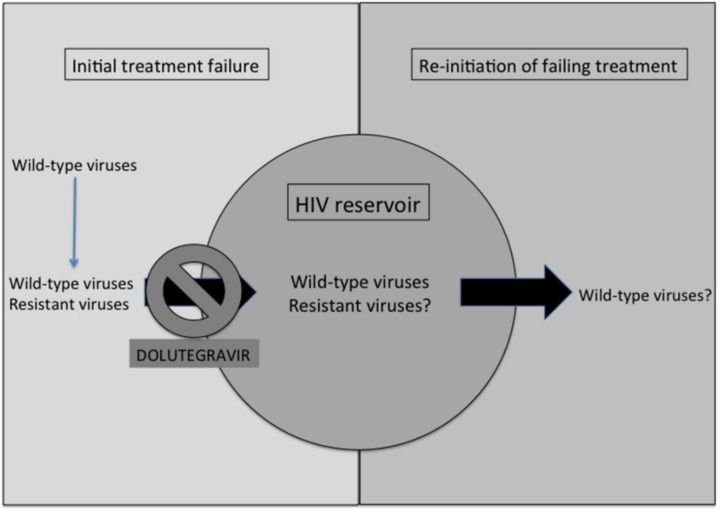
Dolutegravir may inhibit HIV resistant viruses from becoming archived within the viral reservoir.

The unique properties of DTG in regard to drug resistance are a direct consequence of its potency, low nanomolar effective concentrations, favourable pharmacodynamics and ability to bind to the integrase enzyme, and widespread tissue distribution [51,52,53,54,55,56,57]. Interestingly, cerebrospinal concentrations of RAL and DTG have been found to be comparable at approximately 15 ng/mL but effective concentrations of DTG may be superior because of its higher potency compared to RAL [51,58,59]. Integrase inhibitor pharmacokinetics have been recently reviewed elsewhere [59]. In addition, we have shown that DTG maintains more durable inhibitory effects on HIV-1 replication after drug removal than RAL, an observation that correlates with the fact that DTG has a longer dissociative half-life when bound to HIV integrase than does RAL [60]. For example, one treatment-naïve participant in the dose-ranging SPRING-1 clinical trial who was treated with a suboptimal 10 mg dose of DTG and who failed therapy developed the M184M/V substitution in RT [35], showing that the ability of 50 mg DTG to prevent the emergence of drug resistance against the NRTIs that are co-administered with it is dose-dependent. These observations suggest that favourable drug pharmacokinetics and tissue distribution play key roles in the superiority of DTG *versus* other antiretroviral drugs in regard to HIV drug resistance. The only other report of *de novo* resistance in the viruses of individuals treated with DTG in a INSTI-naive setting is from the SAILING clinical trial described above [42]. In contrast with RAL and EVG, the ability of DTG to protect against resistance involving NRTIs suggests that DTG may be superior at inhibiting the replication-competent dynamic component of the HIV reservoir (Figure 2). This argument is supported by the high *versus* low rates of emergent drug resistant viruses in individuals who received monotherapy *versus* HAART, respectively, for treatment of HIV disease. Given the fact that DTG is an INSTI that acts prior to HIV integration and therefore prior to the establishment of long-lived integrated viral reservoirs, we believe that DTG may prevent residual viral replication-competent viruses from becoming part of the viral reservoir (Figure 2). In fact, this hypothesis is currently being evaluated through a registered clinical trial in individuals initiating therapy with DTG as soon as possible after HIV diagnosis (NCT02370979, www.clinicaltrials.gov). Importantly, stable, non-replicating reservoirs will not be affected by treatment intensification with DTG. However, other clinical trials that will include measurements of the size of HIV reservoirs in response to treatment intensification with DTG may be initiated and such trials might have a more positive outcome than those that were performed with RAL [61,62,63,64,65,66,67]. Negative results on the size of the viral reservoir were also reported when both RAL and the CCR5 antagonist maraviroc were used to intensify antiretroviral therapy [68,69,70]. Importantly, the M184I substitution was transiently detected within the HIV integrated DNA from one individual undergoing RAL intensification [71], an observation that confirmed that RAL use does not protect against the emergence of viruses that are resistant against NRTIs. Together with the observation that optimal dose of DTG does protect against the emergence of such viruses, even in individuals failing DTG-based therapy (Table 3), this supports the hypothesis of potential added benefit with DTG intensification.

How is it possible that the occurrence of a replication-competent HIV variant that displays modest resistance to DTG might impact on the HIV reservoir? There is little doubt that the reservoir in almost all HIV-infected individuals consists largely of the viruses that mediated infection in the first place, *i.e.*, wild-type viruses in almost all cases except for people who were initially infected with drug-resistant viruses, in which case it is the latter that will have been archived into reservoirs.

Later in the course of disease, drug-resistant viruses that may have emerged during the course of treatment failure will also become part of the HIV reservoir and this probably includes viruses that become resistant to integrase inhibitors including DTG. However, the ability of the latter viruses to successfully replicate in the absence of drug pressure may be severely compromised relative to both WT viruses and viruses that contain mutations that have a much lower impact on viral replicative capacity. Hence, withdrawal of therapy for reasons that relate to either non-adherence or a treatment interruption would be expected to yield mostly WT viruses and replication-competent drug-resistant viruses but not viruses containing the R263K/H51Y DTG-resistant viruses that have been shown to have vastly diminished replicative capacity and integrase activity. Furthermore, there is a high degree of likelihood that DTG will remain active against the latter viruses because of the very long dissociative half-life between DTG and integrase protein, very tight binding between DTG and integrase, and because the level of resistance against DTG that is conferred by the R263K/H51Y substitutions is relatively slight.

In this context, the reintroduction of DTG as part of a second round of therapy after treatment interruption and/or treatment failure should lead to re-suppression of viral load and, as well, this should result in the killing of both the viruses that may have become activated from reservoirs of long-lived latently infected T-cells as well as actively replicating viruses, regardless whether such viruses are WT or contain mutations associated with drug resistance against any type of ARV. Since the numbers of cells in the body that are capable of serving as hosts of the HIV reservoir must be finitely limited, the use of a drug against which resistance might not be able to occur could change the paradigm of HIV therapeutics and help to point the way forward to a successful eradication strategy.

Of course, this would depend on there being a continuing inability on the part of HIV to generate compensatory mutations that can restore replicative fitness in the aftermath of development of resistance to DTG. It is now almost two years since the approval of DTG by the US Food and Drug Administration (FDA), with no cases of clinical resistance to DTG having yet been reported in the literature in the aftermath of first-time DTG use outside the context of defined clinical trials. Nonetheless, we must be wary that resistance to DTG in such settings may ultimately occur.
